# An equity analysis on the household costs of accessing and utilising maternal and child health care services in Tanzania

**DOI:** 10.1186/s13561-022-00387-7

**Published:** 2022-07-08

**Authors:** Peter Binyaruka, Josephine Borghi

**Affiliations:** 1 Department of Health System, Impact Evaluation and Policy, Ifakara Health Institute, PO Box 78373, Dar es Salaam, Tanzania; 2 Department of Global Health and Development, London School of Hygiene and Tropical Medicine, 15-17 Tavistock Place, London, WC1H 9SH UK

**Keywords:** Equity, Inequality, Direct costs, Time costs, Transport costs, Universal coverage, Healthcare financing, Tanzania

## Abstract

**Background:**

Direct and time costs of accessing and using health care may limit health care access, affect welfare loss, and lead to catastrophic spending especially among poorest households. To date, limited attention has been given to time and transport costs and how these costs are distributed across patients, facility and service types especially in poor settings. We aimed to fill this knowledge gap.

**Methods:**

We used data from 1407 patients in 150 facilities in Tanzania. Data were collected in January 2012 through patient exit-interviews. All costs were disaggregated across patients, facility and service types. Data were analysed descriptively by using means, medians and equity measures like equity gap, ratio and concentration index.

**Results:**

71% of patients, especially the poorest and rural patients, accessed care on foot. The average travel time and cost were 30 minutes and 0.41USD respectively. The average waiting time and consultation time were 47 min and 13 min respectively. The average medical cost was 0.23 USD but only18% of patients paid for health care. The poorest and rural patients faced substantial time burden to access health care (travel and waiting) but incurred less transport and medical costs compared to their counterparts. The consultation time was similar across patients. Patients spent more time travelling to public facilities and dispensaries while incurring less transport cost than accessing other facility types, but waiting and consultation time was similar across facility types. Patients paid less amount in public than in private facilities. Postnatal care and vaccination clients spent less waiting and consultation time and paid less medical cost than antenatal care clients.

**Conclusions:**

Our findings reinforce the need for a greater investment in primary health care to reduce access barriers and cost burdens especially among the worse-offs. Facility’s construction and renovation and increased supply of healthcare workers and medical commodities are potential initiatives to consider. Other initiatives may need a multi-sectoral collaboration.

## Introduction

Many developing countries are working to attain the universal health coverage (UHC) goal by 2030, which states that everyone needs to access good quality health care without incurring any financial hardship due to health care payment [1]. Underpinning UHC is equity in financing, accessing, and using health care services [2]. Financing equity implies that payment for health care should be based on ability to pay, and equity in access implies that benefits from health care should be based on need regardless of individual background characteristics [2–4]. To date, the poorest in low-income countries are often constrained in accessing and utilising health services, including maternal and child health (MCH) services [5, 6]. For instance, a study across 54 low income countries revealed that coverage of skilled birth attendants was only 32% in the poorest quintile, compared with 84% in the richest quintile [5]. Financial cost is one of the barriers that prevent the poorest from accessing care [7–9]. They incur costs either directly by spending on transport and medical care costs, or indirectly through time spent accessing care, which may result in income loss from being unproductive [10]. These costs are against the UHC goal as they pose financial risks and can push households into poverty [1, 10, 11].

The two measures of financial protection for UHC, catastrophic expenditure and impoverishment effect, only consider the degree of financial protection in relation to direct medical costs [1, 12]. Most prepayment schemes focus on protecting people against medical costs associated with health care, typically ignoring transport or travel costs [1, 13]. Analyses of health financing progressivity also focus on the distribution of direct medical payments and contributions to prepayment schemes across socioeconomic groups [14–16]. To date, other aspects of direct costs (e.g., transport costs) and indirect costs (e.g., time/ opportunity costs) have not been considered within equity analyses or in monitoring progress on financial protection towards UHC.

However, some evidence shows that time and transport costs can equally limit access to health care and sometimes even more so than direct medical costs [7–9, 17–19]. For instance, patients may not seek care due to time costs of accessing care, due to distance or long waiting times at facilities [7, 20]. Transport cost can also contribute to catastrophic expenditure [11], and sometimes represent a significant share of total out-of-pocket payments for health care [21–23]. When valued based on income foregone, time costs varied from 9 to 73% of total household cost of care seeking [23, 24]. Travel and time costs are significant for obstetric care, particularly when complications arise [19, 25–27]. A study in Tanzania [28] revealed that transport costs were almost half of total expenditure for a normal delivery, while travel and waiting time were estimated at 65–93% of total household expenditure for a delivery. Given the potential significance of time and travel costs, it is equally important to assess their distribution across population subgroups to understand the equity implications of these costs in the premise of leaving no one behind towards UHC.

To date, the studies in Tanzania assessing the distribution of costs across population subgroups, have focused on direct medical expenditures [29–32] and catastrophic spending for health care [33], only a few studies have examined the distribution of transport and time costs [28, 34, 35]. However, the studies on transport and time costs in Tanzania are either outdated and rural focused (e.g. [28]), or had a narrow geographic coverage of one district (e.g. [34, 35]); but also did not compare different dimensions of time cost, or examine the distribution of these costs across population subgroups, facility and service types.

This study, therefore, estimated time costs as well as transport and medical costs of accessing and utilising MCH services; explored the main driving cost items by comparing costs across facility types and MCH service types; and examined the distribution of costs across patient socioeconomic groups and place of residence (urban/rural) in Tanzania. This assessment provides evidence to policy makers on the areas that need health sector/ multisectoral interventions in order to improve access and use of primary and essential MCH services in Tanzania.

## Methods

### Study setting

Tanzania is a lower middle-income country in East Africa with an estimated population of around 56 million people in 2016 [36]. Tanzania has 31 regions and most (70%) inhabitants are residing rural areas. Tanzania has made progress on child survival, with little improvement in maternal health [37, 38] and this is regarding the Millennium Development Goals (MDGs) of reducing by two-thirds of child mortality and by three-quarters maternal mortality ratio. In particular, over the past 15 years from 1999 to 2015/16 in Tanzania, the infant and under-5 mortality rates have declined from 99 deaths to 43 deaths per 1000 live births and from 147 to 67 deaths per 1000 live births, respectively [37]. The maternal mortality ratio also declined from 578 deaths to 454 deaths per 100,000 live births in 2004/5, before raising up to 556 deaths in 2015/16 [37, 39]. Access to one antenatal care (ANC) is almost universal, but there remains relatively low coverage of at least four ANC visits (51%), institutional delivery (63%) and postnatal care (PNC) (33%) [37]. The use of maternity services shows a marked imbalance along the continuum of care as reported elsewhere [40–42]. Also, 75% of Tanzanian children age 12–23 months received all basic vaccinations [37].

The health system in Tanzania involves a predominance of public sector facilities, followed by faith-based providers, and a limited number of private-for-profit providers. The public health system has a hierarchical administrative structure, with a referral structure such that dispensaries, health centres, and district hospitals provide primary health care (PHC) services. A dispensary is supposed to serve at least one village, and a ward for a health centre [43]. Tanzania implemented a Primary Health Service Development Programme (2007–2017) to improve access to basic health care service by rehabilitating and constructing at least one dispensary per village and a health centre per each ward countrywide [43].

The health financing system in Tanzania is highly fragmented with many sources including general taxation (34%), donor support (36%), out-of-pocket payments (22%), and health insurance contributions (8%) [44]. In 2018, the health sector review revealed that 33% of Tanzanians are covered by health insurance, which include 8% by National Health Insurance Fund (NHIF) for public servants mainly, 25% by improved Community Health Fund (iCHF) for people working in informal sector, and 1% by private insurance and Social Health Insurance Benefit (SHIB) [45]. The coverage of health insurance is still low, which exposes many Tanzanians to financial risks due to direct health care payments. Despite exemption and waiver policies in Tanzania which aim to protect poor and vulnerable groups (e.g., pregnant women, children, and elders) [46, 47], the enforcement of these policies is weak [29, 48]. Also, the existing health insurance schemes in Tanzania only cover medical expenses at facilities, but do not compensate patients for travel and time costs incurred when accessing care.

### Data

Data were collected from a cross-sectional survey of patients from three regions (Pwani, Morogoro and Lindi) in Tanzania. All seven districts of Pwani region and four districts from Morogoro and Lindi region were included. This study was part of the large baseline survey of an impact evaluation of a pay for performance (P4P) programme in Pwani region [49, 50]. The evaluation study used Morogoro and Lindi as comparison regions. A sample of 75 facilities from Pwani region were considered and the same number from comparison districts, including hospitals (*n* = 6), health centres (*n* = 16) and dispensaries (*n* = 53) in each arm. Comparison facilities had similar levels of outpatient care visits and staffing levels to intervention facilities. In total,150 public and private health facilities (12 hospitals, 32 health centres and 106 dispensaries) were surveyed (82% were public facilities). Data were collected through patient exit-interviews to a maximum sample of 10 clients/ patients per facility between January and February 2012. Clients were approached upon arrival at the facility, asked a series of screening questions to check their eligibility. Eligible respondents included those resided in that area for at least 6 months, aged at least 18 years, and seeking care for one of the following four services: (i) ANC, (ii) child vaccination for under 1 year, (iii) PNC follow-up for mothers/ babies 2 months after birth, and (iv) check-up for fever, cough and diarrhoea for women/ under 5 children. Thus, respondents included pregnant women, mothers or care givers who brought under 5 children to the facility. Prior to the interview, all eligible clients were asked for their consent to participate in the survey after exiting the consultation room. The exit-interview tool was adapted from the World Bank Impact Evaluation Toolkit [51], which measured a range of quality-of-care indicators including patient satisfaction/ experience of care, and costs of accessing and utilising health services. The exit-interviews also captured information on household background characteristics (e.g., ownership of assets and housing characteristics) that were used to assess the household’s socioeconomic status. All the interviews were conducted in Swahili language. A tool was pre-tested for consistency, relevance, and clarity before the actual survey.

### Outcome variables

The outcome of interest includes time costs as well as transport and medical costs. Time costs were estimated in minutes associated with traveling to and waiting or receiving consultation at the facility. Transport and medical costs were measured in local currency, Tanzanian shilling (TZS), and then converted into US dollar (USD) using the approximate exchange rate during the survey in 2012 (1 USD equal 1600 TZS). All costs were estimated based on patient recall. Transport and time costs of travelling were measured for one-way journey to the health facility. We ‘multiplied by two’ for simplicity to account for a return trip, since patients on their return sometime pass via markets or to other social activities as previously reported in Tanzania [34]. In order to avoid overestimation, the one-way journey is preferred. For robustness check, however, we presented the estimates for both one and two-way travel costs.

### Equity dimensions

We examined the distribution of time and direct costs by two dimensions of equity – (i) place of residence (rural/urban) and (ii) household’s socioeconomic status (quintiles). The rural-urban dimension was considered to reflect the remoteness and how facilities are scattered, which has important implications for transport costs and travel time; while the socioeconomic status was included to measure the households’ living standard as a proxy of ability to pay. Household socioeconomic status was assessed through a wealth index based on household characteristics and asset ownership derived using principal component analysis based on 42 items (Appendix Table 5 & 6) [52, 53]. Patients were ranked by wealth scores from poorest (low score) to least poor (high score), and classified into five equal-sized quintiles.

### Data analysis

We first described the mean and median costs by patient socioeconomic status and residence. The equity analyses proceeded by using three measures of inequality –an absolute measure (the gap) and two relative measures (the ratio and the concentration index) [15, 54]. The equity gap was measured as the difference in costs between patient subgroups, while the equity ratio was measured as the ratio of costs between patient subgroups. Specifically, both equity gap and equity ratio were calculated between poorest and least poor patients, as well as between rural and urban patients. When comparing the poorest and least poor patients, for example, a positive (negative) gap and a ratio greater (less) than one defines high-cost burdens among the poorest (least poor), respectively. A gap of zero or a ratio of one defines an equal distribution in costs. We also used t-tests to assess whether the gaps were significantly different from zero.

In addition, we computed the concentration index (CI) to quantify the degree of socioeconomic-related inequality in cost burdens of seeking and receiving health care. The CI was computed on a ranking variable of household socioeconomic status as shown in Eq. (1) [15, 55].

1 $$CI=\frac2\mu cov{(y_i,R_i)},$$

where *y*_*i*_ is the cost variable of the *i*^*th*^ patient; *R*_*i*_ is the fractional rank of the *i*^*th*^ patient (in terms of households’ socioeconomic status, with lower fractions for poorest and larger fractions for richest); *μ* is the average cost and cov denotes the covariance. The CI ranges between [− 1 and + 1], whereby zero indicate equality between socioeconomic status subgroups, while negative and positive values indicate that poorest have high-cost burdens and low-cost burdens, respectively. We also tested whether the CIs were significantly different from zero.

As a robustness check, our analysis was also restricted to public facilities (82%) as these facilities are supposed to offer free MCH services in Tanzania. All analyses were performed using STATA version 16.

## Results

### Characteristics of respondents and facilities

A total of 1407 patients from 150 health facilities participated in exit-interviews. Most patients who were interviewed were seeking care for children under 5 years (39.6%), while childhood vaccination was sought by the least clients (9.7%) (Appendix Table 7).

Most facilities visited were public owned, dispensaries, and had a staffing level of 17 health workers on average (Table 1). Patients were mostly (82.8%) residing in rural districts. A majority of respondents were married (68%), farmers (59.7%), Muslim (73.2%), and with at least primary education (72.4%). Few respondents (9%) were from a household with a health insurance (Table 1).

**Table 1:** Descriptive statistics of facility and patients’ characteristics (*n* = 1407)

Characteristics	Description	Mean [SD]	Mean in %
**Panel A: Facility characteristics**			
Facility ownership	=1 for public owned		81.9
Facility level of care	=1 for dispensary		67.7
Staffing level	Number of staff	17.2 [32.5]	
**Panel B: Clients’ individual and household characteristics**			
Age of woman	Maternal age (15–49) years	26.8 [7.0]	
Age of a child	Child age in months	15.6 [13.4]	
Marital status	=1 for married woman		68.0
Education	=1 for primary education/above		72.4
Occupation	=1 for farming activities		59.7
Religion	=1 for Muslim woman		73.2
Household size	Number of household members	5.9 [3.0]	
Health insurance status	=1 for any insurance at household		8.9
Place of residence	=1 for rural district resident		82.8
Household socioeconomic status (SES)			
SES –quintile 1	=1 for poorest household		20.0
SES –quintile 2	=1 for poor household		19.9
SES –quintile 3	=1 for middle wealth household		20.0
SES –quintile 4	=1 for less poor household		19.9
SES –quintile 5	=1 for least poor household		19.9

Reference categories of facility type include public vs. non-public facility, dispensary vs. health centres and hospitals

*SD* Standard Deviation

### Time costs by socioeconomic status and place of residence

**Fig. 1:**
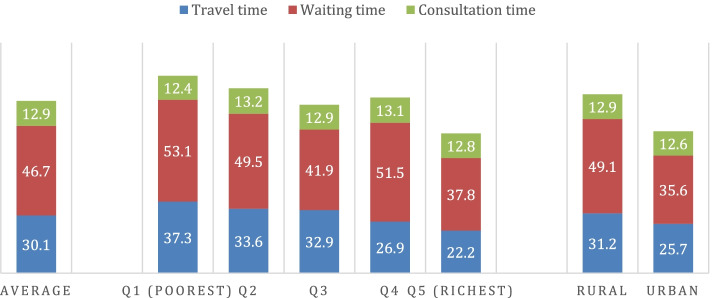
Time costs of accessing and utilising health services (in minutes)

We found most patients (71%) accessed care on foot. The use of car, motorcycle or bicycle to access care was pro-rich while travelling on foot was pro-poor (Table 2). Figure 1 shows the average time costs of accessing and using health care and its distribution, while Appendix Table 6 shows the associated median value.

**Table 2:** Direct and indirect costs of accessing and utilising health care by socioeconomic status quintiles and place of residence

			Socioeconomic status Equity measures			Place of residence Equity measures	
	n	Mean	Gap (Poorest -least poor)	Ratio (Poorest/ least poor)	Concentration Index (CI)	Gap (Rural - Urban)	Ratio (Rural/ Urban)
**Mode of transport**	1407	(1)	(2)	(3)	(4)	(5)	(6)
Foot (%)	(*n* = 1000)	71.1	22.1***	1.4	−0.069***	4.0	1.1
Car (%)	(*n* = 119)	8.5	−7.5**	0.4	0.221***	−6.7	0.5
Motorbike & bicycle (%)	(*n* = 288)	20.5	−14.7***	0.5	0.152***	2.7	1.1
**Time cost (mins)**							
Travel time for all	1143	30.1	15.1***	1.7	−0.109***	5.5*	1.2
*Foot*	(*n* = 804)	31.3	14.7***	1.6	−0.104***	5.3	1.2
*Car*	(*n* = 96)	26.7	1.1	1.0	−0.019	0.3	1.0
*Motorbike & bicycle*	(*n* = 243)	27.8	17.3**	1.9	−0.141****	8.4**	1.4
Waiting time	1394	46.7	15.3***	1.4	−0.056**	13.5**	1.4
Consultation time	1374	12.9	−0.4	1.0	0.002	0.3	1.0
**Direct cost (USD)**							
Transport cost for all clients	1299	0.41	−0.51***	0.2	0.269***	−0.23*	0.6
*Car*	(*n* = 119)	1.32	− 0.99*	0.5	0.184	−0.87	0.6
*Motorbike & bicycle*	(*n* = 267)	1.39	−0.75***	0.4	0.065*	−0.31	0.8
Prob. of paying for medical care (%)	1399	17.8	−9.3***	0.6	0.128***	1.4	1.1
Medical cost for all clients	1399	0.23	−0.32***	0.2	0.288***	−0.16	0.6

The median travel time overall =20 min, while on foot (21.5 min), car (20 min) and other modes (20 min); median travel cost = 0 USD; median waiting time = 26.5 min, and consultation time = 10 min; median medical cost = 0 USD; travel time reflects a one-way journal

*** denotes significance at 1%, ** at 5%, and * at 10% level

The average and median **travel time** to reach a facility were 30.1 minutes and 20 minutes, respectively. The burden of travel time was significantly dominated among the poorest and rural patients (Fig. 1). These patterns were supported by the positive equity gaps (15.1 min), negative concentration index (− 0.109), and equity ratios greater than one (1.7) (Table 2). The average **waiting time** and **consultation time** were 46.7 minutes and 12.9 minutes, respectively (Fig. 1). Poorest and rural patients waited more with an equity gap of 15.3 min and 13.5 min, respectively, than their counterparts; while consultation time was not significantly different across quintiles and place of residence (equity gaps around zero) (Table 2).

The total time cost of accessing and receiving health care were 90 minutes on average (120 minutes including return trip). This total time cost was driven by waiting time (52%) followed by travel time (33.6%) with relatively few minutes for consultation (Appendix Fig. 3). However, when considering total travel time including the return trip, the travel time takes the largest share of time costs (50.3%) followed by waiting time (38.9%) and consultation time.

### Direct costs by socioeconomic status and place of residence

**Fig. 2:**
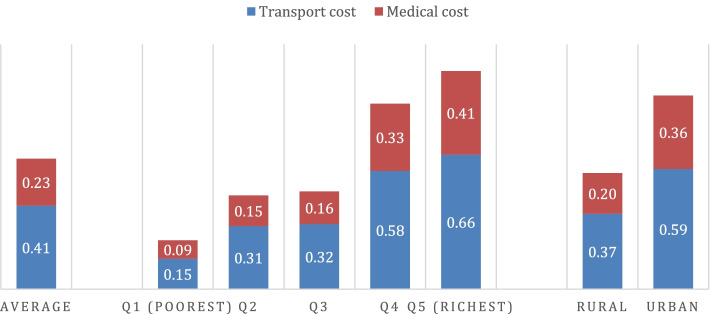
Direct costs of accessing and utilising health services (in USD)

Figure 2 shows the direct transport and medical costs and their distributions, while Appendix Table 6 shows the associated median values. The average transport cost for one-way to reach a facility was 0.41USD (the median value equals zero due to high degree of skewness, Appendix Table 6), although only 21.3% paid for transportation costs. The cost burden on transport was significantly higher among the least poor (0.66USD vs. 0.15USD) and among urban patients (0.59USD vs. 0.37USD) than their counterparts (Fig. 2). Consistently, the associated equity gaps were negative (− 0.51), with a positive concentration index (0.269) and equity ratios less than one (0.2) (Table 2).

In terms of medical cost, 17.8% of patients paid out-of-pocket for health care at the point of use. On average, the medical cost incurred across all patients was around 0.23USD. However, the likelihood of paying and the average amount paid were greater among the least poor patients (0.41USD vs. 0.09USD) and among urban residents (0.36USD vs. 0.20USD) than their counterparts (Fig. 2).

In terms of cost share, the transport cost took a larger share of direct cost. Specifically, transport costs to access care took almost two-thirds of total direct cost (64.1%) and more than three-quarter of total direct cost (78.1%) when including the cost for a return trip (Appendix Fig. 4).

Table 3 shows the distribution of time and direct costs by facility ownership and level of care. Patients who accessed public facilities and dispensaries spent significantly more time travelling, about 31.6 min and 31.9 min, respectively; but incurred less transport cost about 0.32USD and 0.33USD respectively than those who accessed non-public facilities and hospitals (Table 3). Waiting and consultation time was similar across facility types. As expected, due to free MCH services in public facilities, patients were significantly less likely to pay for care in public facilities (13%) than in non-public facilities, and also paid significantly less amount 0.07USD than in non-public facilities (Table 3).

**Table 3:** Time and direct costs by facility ownership and level of care

Cost variable	N	Mean	Facility ownership			Facility level of care		
			Public/ government	FBO	Private/ other	Hospital	Health Centre	Dispensary
	1407	(1)	(2)	(3)	(4)	(5)	(6)	(7)
**Time costs (mins)**								
Travel time for all clients	1143	30.1	31.6 (ref)	25.5**	22.3***	25.3 (ref)	26.9	31.9***
Waiting time	1394	46.7	48.3 (ref)	39.5*	39.8	59.3 (ref)	45.9	45.1
Consultation time	1374	12.9	12.9 (ref)	12.8	13.4	13.5 (ref)	14.2	12.4
**Direct cost (USD)**								
Transport costs for all clients	1299	0.41	0.32 (ref)	0.76***	0.95**	0.69 (ref)	0.52	0.33***
Prob. Of paying for care (%)	1399	17.8	13.0 (ref)	49.2***	17.1	19.3 (ref)	8.9	20.5
Medical cost for all clients	1399	0.23	0.07 (ref)	1.14***	0.42	0.46 (ref)	0.11*	0.23

t-test used (public facility and hospital) as reference groups; travel time reflects a one-way journal

*** denotes significance at 1%, ** at 5%, and * at 10% level

Table 4 shows the distribution of time and medical costs by types of MCH service sought. ANC patients spent significantly longer time waiting (56.2 min) and in consultation with providers (16.3 min) than those seeking other services (Table 4). The probability of paying for medical care did not vary by service types. However, PNC and vaccination clients paid less around 0.06USD and 0.04USD, respectively than ANC clients, while check-up clients paid more than ANC clients (0.43USD vs. 0.17USD) (Table 4).

**Table 4:** Time and medical costs by service type sought

Cost variable	*N* = 1407	Mean	Service type sought			
			ANC	PNC	Vaccination	Check-up
			(*n* = 334)	(*n* = 380)	(*n* = 136)	(*n* = 557)
**Time cost (mins)**						
Waiting time (min)	1394	46.7	56.2 (ref)	39.1***	38.6**	48.1
Consultation time (min)	1374	12.9	16.3 (ref)	9.4***	10.3***	13.8***
**Medical cost (USD)**						
Prob. Of paying for care (%)	1399	17.8	13.5 (ref)	10.3	10.3	27.4
Medical cost for all clients	1399	0.23	0.17 (ref)	0.06**	0.04**	0.43***

t-test used ANC service as a reference group; PNC is for follow up mothers and under 2 months babies after delivery; Check-up is for self/ under 5 child check-ups for fever, cough and diarrhoea

*** denotes significance at 1%, ** at 5%, and * at 10% level

We further restricted the analysis to public facilities because of free MCH services in public facilities. This restriction increased slightly the average time costs (except consultation time as it remained around 12.9 min) while average direct costs decreased (0.07USD) (Appendix Table 9). The equity results remained almost unchanged between patients’ subgroups.

## Discussion

This study estimated time costs as well as transport and medical costs of accessing and utilising MCH services at PHC facilities, and examined the distribution of these costs across patient subgroups. This study adds to a limited evidence base examining transport and time costs and examining their equity in a LMIC setting. An advantage of the study is the use of patient exit-interviews to minimise recall bias. We found that overall, the time cost associated with seeking outpatient care was 90 minutes on average, driven primarily by travel and waiting time. The burden of travel and waiting time were significantly greater for the poorest groups, while consultation time was similar across wealth groups; waiting time was also significantly higher among rural compared to urban respondents. In terms of direct costs, transport costs were almost double compared to medical costs, with a large majority not facing medical costs associated with care seeking. The burden of transport and medical expenditures were significantly higher among the least poor and among urban respondents. Patients spent more time travelling to public facilities and dispensaries than other provider types, but waiting and consultation time did not vary significantly by facility types. Patients were less likely to pay for care in public facilities, and ANC clients faced the longest waiting and consultation times.

Our estimate of travel time, half an hour on average, is similar to a previous study in Tanzania [28], but lower than a study in Malawi which estimated a median 1 hour travel time to a health centre [18]. However, our estimate of 1.9 USD for transport cost among those who paid something (0.4 USD for all clients) is lower than the 2.5 USD previously reported in Tanzania [32]. Our finding that the poorest and rural patients faced significant time burden accessing care and paid less on transport cost is largely explained by the means of transport, since the poorest and rural patients often travel on foot. In many settings including Tanzania, lower-level public facilities such dispensaries are much preferred by poorest and rural patients as their closest facilities and they offer ‘free’ PHC services [16, 20, 30].

Patients in our sample spent on average 47 minutes waiting for MCH services, 56 minutes for ANC only, which is less than previously reported (1 hour and half) for ANC in Tanzania [34]. Our analysis revealed that the poorest and rural patients waited longer than their counterparts which is consistent with the pattern observed across hospitals in high income countries [56–58]. Ours is the first study to reveal this evidence from a LMIC. However, the waiting time in high income countries is measured as number of days passed from the date a patient was added in the waiting list and the date of actual admission for treatment. In our setting, the longer waiting time to enter into the consultation room especially among the poorest and rural patients indicates the inadequate supply of health care –including shortage or maldistribution of health facilities and human resources for health [20]. Tanzania like other developing countries has significant shortage of health staff with relatively more staff (e.g., specialists) in urban settings [38, 59]. The least poor patients also waited for shorter time possibly because majority of them are able to pay informal payment or ‘under the table’ to health workers in order to jump the queue [60].

Our study estimated about 16.3 minutes consultation time for ANC, which is slightly higher than 15 minutes reported previously in southern Tanzania [35], higher than 10 minutes reported previously in Dar es Salaam [61], but less than 20 and 48 minutes reported earlier in rural Ngorongoro district [62] and in Kisarawe district for a mobile clinic [34], respectively. However, with the exception of consultation time reported in Kisarawe Tanzania [34], the other estimates fall below the recommended time between 30 and 40 minutes for ANC particularly first visit [63]. The consultation time in our study was generally similar across subgroups of patients. Our 13 minutes of consultation time for MCH services is relatively longer compared to approximately 5 minutes for outpatient consultation reported in Mozambique [64] and Nigeria [65]. Since medical doctors and clinicians use longer consultation time than nurses and midwives [61], there is a need for qualified staff to offer comprehensive consultation to clients.

The finding that shows patients spent on average more time on waiting than consultation is consistent with findings from previous studies in Tanzania for ANC [34] and elsewhere for outpatient consultations [64, 65]. This is partly explained by the persistent shortage of healthcare workers and health facilities especially in rural settings [65–67]. We further found that services with shorter consultation time (e.g., PNC and vaccination) also had shorter waiting time. This implies that consultation time plays a significant role in explaining how long patients would wait for health care.

The total time cost of accessing and using MCH was largely driven by waiting time as previously reported in Tanzania [28, 34]. In our study, the waiting time contributed almost 52.1% of total time cost, and about 53% when ANC service only considered. This is similar to previous studies that found half of the total time cost were spent waiting for ANC services in Tanzania [28, 34]. However, when including the time spent travelling back home, travel time became the main contributor of total time cost. In terms of share for total direct cost, the main driver was transport cost which contains 64% of total costs, although it was less than a dollar on average. This finding is consistent to what reported earlier in southern Tanzania, where travel costs to access maternity services represented almost a half of total direct costs [28]. A similar pattern was reported in Nepal [23], where transport cost took more than 50% of the total costs for clients seeking delivery care. However, in Bangladesh [68] and Nigeria [69] transport cost took relatively lesser share of about 20 and 32% of total costs for maternity services, respectively. Also, transport cost accounted for 42% of health expenditure in South Africa [70]. Our results imply that transport cost contribute significantly to total health care costs, and have the potential to deter individuals from accessing health care especially among the poorest and those residing in remote areas.

Moreover, we found almost 18% of women paid out-of-pocket for MCH services in Tanzania. When restricting the analysis to public facilities (82% in our sample), about 13% paid out-of-pocket for MCH services that are supposed to be offered free of charge in Tanzania. However, the likelihood of paying for MCH service was significantly lower in public compared to private facilities. Since our sample included only patients at the facility level, it is likely that the extremely poorer never accessed health facility due to financial barriers, and possibly our results are reflecting the least poor and near poor patients only. Unsurprisingly, the direct payments for exempted MCH services have been reported before in Tanzania [29, 46, 48] and elsewhere [68–71]. Paying for services which are exempted indicates a limited financial protection and weak enforcement of the exemption policy in Tanzania [46, 48]. The inadequate budget allocation to the health sector [48, 72] possibly affecting the enforcement of the exemption policy. In LMICs, however, expanding the resource envelope for health is constrained by limited fiscal space, inefficient in revenue collection [73] and the larger share of people in the informal sector who hardly enrol into prepayment mechanisms [74, 75].

Our findings have important policy implications. Prepayment mechanisms and user fee removal are important steps towards UHC [1], but do not guarantee health care access due to other barriers such as transport and time costs which are often neglected [8, 9]. Our findings indicate that the poorest and rural patients faced a relatively greater cost burden in terms of time lost from productivity partly because they have limited ability to pay for transport and/or health care; while their counterparts incurred huge direct cost because they have the ability to pay for transport and/or health care. Since the worse-off patients spent more time travelling, mostly on foot, to access public and lower level facilities, this reinforces the need for a greater investment in PHC facilities in order to bring quality health services closer to the population as one of the recommended routes toward UHC [76]. This can be through PHC facility’s construction and renovation and an increase in the supply of healthcare workers and medical commodities. These initiatives may help to reduce the time and direct costs of accessing and using PHC especially in remote and rural areas. Investing in PHC facilities will also meet patients’ needs and expectations and eventually reduce the time and travel costs incurred by patients bypassing closer PHC [77, 78]. In support of that argument, Tanzania implemented a Primary Health Care Services Development Programme (PHSDP) from 2007 to 2017 which involved construction and renovation of PHC facilities [43, 79]; and interestingly, the current government phase is continuing with construction and renovation of PHC facilities. Investing in PHC facilities also aligns with the Alma Ata Declaration on PHC in 1978 [80] and the Astana Declarations of 2018 [81] for the purpose of achieving health for all and the UHC goal [76]. Future research should examine the effect of investing in PHC on time and direct costs of access and using health care.

Our findings highlight the need for LMIC policy makers to consider the potential expansion of health insurance benefit packages to cover transport costs, rather than covering medical costs only. Other approach to reduce the travel time especially among the worse-offs is improving access to means of transportation though would not necessarily affect the transport costs [82]. The success of this approach depends on other sectors beyond the health sector (e.g., transportation and infrastructure sector), which indicates the need of a multisectoral approach to reduce access/ geographical barriers. Further evidence suggests potential initiatives to reduce the costs of accessing care such as conditional cash transfers [83], vouchers to cover transport costs [84, 85], expanding outreach services (e.g. mobile clinics) [9], establishing maternity waiting homes [86] and implementing targeted policies for vulnerable and remote populations [87, 88]. However, some of the suggested strategies are costly and may need strong political will and a multisectoral collaboration.

This study has some strengths. First, we studied time costs of accessing and using MCH services and transport costs as one of the cost aspects that received less attention despite its potential to limit health care access and use. Second, our time cost reflected a wider spectrum including time travelling, as well as waiting and consultation time. Third, this study examined the distribution of time and direct costs with equity implications. This is an important assessment as it shows who bears the cost burdens as an entry point for intervention. Fourth, we explored how time and direct cost varied by facility and service types, since previous studies largely focused on either one facility or service type. Lastly, we collected data through patient exit-interviews as an approach to reduce the recall bias as they had a recall period of less than 24 hours.

However, our study had some limitations. First, we were unable to assess the affordability of the amount paid or to undertake financing incidence analysis due to the lack of data on household income/ expenditure to reflect ability to pay. Second, we were unable to explore different coping strategies to finance costs of access and use due to data limitation. Third, we were unable to value the time costs (minutes) into monetary values because of the lack of income data from our sample. There is also considerable variation in measuring and valuing time lost into monetary terms, in some cases varies by age, gender, location or economic activity [10, 89]. Our sample also combined different service types which limits the process of valuing time lost. Fourth, although exit-interviews may have reduced recall bias, our findings reflect only those who were able to access and use health care. Fifth, we used data from 2012 patients’ survey, because these data included different aspects of costs (direct and indirect/time costs) which are lacking in more recent publicly available patient/household survey data. Lastly, we were unable to capture the hospitalisation costs for inpatient clients which adds significantly to cost burden, because of the survey design that focused on assessing quality of care for patients exiting after consultation.

## Conclusion

This study highlights the importance of assessing time and transport costs alongside medical costs when evaluating health care access and use, and financial protection towards UHC in LMIC. Travel costs and waiting time were the main drivers of total cost for outpatient MCH care in the Tanzanian context. The actual total costs of accessing health care are underestimated when taking narrow focus on medical cost alone, since time and transport costs are critical cost drivers in many settings. Future research intending to assess health care access and financial protection should incorporate time and transport costs as well as medical costs, and assess the coping mechanisms and level of affordability of various cost components. Our findings also reinforce the need for policy makers in LMIC to invest more on improving PHC facilities, as a way to reduce the time and cost burdens of accessing and using PHC services especially among the poorest and rural patients. This can be through facility’s construction and renovation and increased supply of healthcare workers and medical commodities. However, efforts to reduce other access barriers may need multisectoral collaboration.

## Figures and Tables

**Fig. 3:**
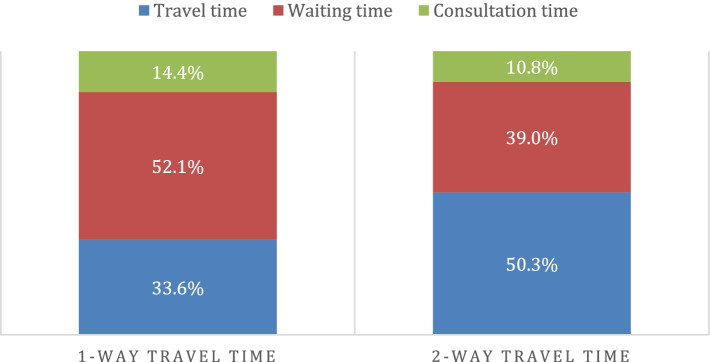
Share of overall time costs

**Fig. 4:**
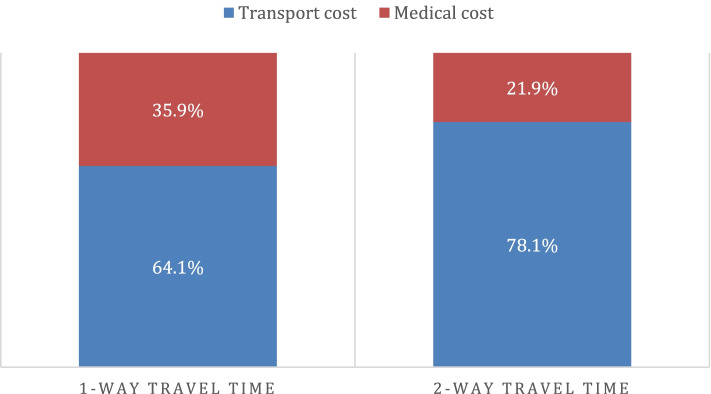
Share of overall direct costs

## Data Availability

The data have been uploaded into a data repository. The DOI URL for the dataset is: 10.5281/zenodo.21709.

## Appendix

Fig. 3

Fig. 4

Table 5

Table 6

Table 7

Table 8

Table 9

**Table 5:** Items used to construct household wealth status score

No.	Variable description
1.	Asset: electricity
2.	Asset: working radio
3.	Asset: working television (TV)
4.	Asset: working DVD
5.	Asset: working mobile phone
6.	Asset: working landline phone
7.	Asset: working iron
8.	Asset: working refrigerator
9.	Asset: working wall watch
10.	Asset: sewing machine
11.	Asset: table
12.	Asset: sofa coach
13.	Asset: cupboard
14.	Asset: motorcycle
15.	Asset: car
16.	Household member with a bank account
17.	Number of sleeping rooms
18.	Source of drinking water: piped water
19.	Source of drinking water: borehole/ covered well
20.	Source of drinking water: open well
21.	Source of drinking water: spring water
22.	Source of drinking water: river/ dam/pond/lake
23.	Toilet type: flush toilet
24.	Toilet type: pit latrine
25.	Toilet type: no/ no/ another toilet
26.	Source of cooking energy: electricity
27.	Source of cooking energy: kerosene/paraffin
28.	Source of cooking energy: charcoal
29.	Source of cooking energy: firewood
30.	Source of light: electricity
31.	Source of light: solar
32.	Source of light: kerosene/ paraffin
33.	Source of light: candle/ firewood
34.	Source of light: torch or another source
35.	Floor material: sand/earth/dung
36.	Floor material: cement
37.	Floor material: other
38.	Wall material: grass/poles/mud wall
39.	Wall material: bamboo with mud wall
40.	Wall material: sundried/ burnt bricks
41.	Wall material: cement blocks
42.	Wall material: stones with mud

**Table 6:** Asset ownership and household characteristics for socioeconomic status scores

Variable	Mean
Electricity	0.136
Radio	0.643
Television	0.112
Telephone	0.669
Wall watch	0.130
Sofa coach	0.243
Cupboard	0.185
Motorcycle	0.077
Car	0.013
Household features	
Piped source of water	0.154
Flush toilet	0.087
Pit latrine toilet	0.854
Charcoal for cooking	0.313
Firewood for cooking	0.659
Kerosene for lighting	0.785
Electricity for lighting	0.129
Sand floor	0.609
Cement floor	0.377
Metal roof	0.585
Mud wall	0.299

**Table 7:** Type of service sought by clients

MCH service types	N = 1407
Antenatal care (ANC)	334 (23.7%)
Postnatal care (PNC) –mother/ baby under 2 months	380 (27.0%)
Postnatal care (PNC) –check-up (self/ baby under 5 years)	557 (39.6%)
Child vaccination for under 1-year children	136 (9.7%)

PNC for follow up mothers and under 2 months babies after delivery; Check-up for self/ under 5 child check-ups for fever, cough and diarrhoea

**Table 8:** Average and median costs of health care access and use

	N	Mean [SD]	Median [IQR]
**Time costs (mins)**			
Travel time for all clients	1143	30.1 [36.9]	20 [10–30]
Waiting time	1394	46.7 [55.6]	26.5 [10–60]
Consultation time	1374	12.9 [11.2]	10 [5–15]
**Direct cost (USD)**			
Transport cost for all clients	1299	0.41 [1.2]	0
Medical cost for all clients	1399	0.23 [0.93]	0

*SD* standard deviation, *IQR* interquartile range [25–75%]

*** denotes significance at 1%, ** at 5%, and * at 10% level

**Table 9:** Direct and indirect costs of accessing and utilising health care by socioeconomic status quintiles and place of residence –for public facilities only

			Socioeconomic status Equity measures			Place of residence Equity measures	
	n	Mean	Gap (Poorest -least poor)	Ratio (Poorest/ least poor)	Concentration Index (CI)	Gap (Rural - Urban)	Ratio (Rural/ Urban)
	1153	(1)	(2)	(3)	(4)	(5)	(6)
**Time cost (mins)**							
Travel time for all	911	31.6	12.7***	1.5	−0.085***	3.5	1.1
Waiting time	1141	48.3	16.5***	1.4	−0.052*	14.3**	1.4
Consultation time	1125	12.9	−1.0	0.9	0.007	1.2	1.1
**Direct cost (USD)**							
Transport cost for all clients	1063	0.32	−0.36***	0.3	0.257***	−0.11	0.7
Medical cost for all clients	1146	0.07	−0.04	0.6	0.153**	−0.04	0.6

The median travel time overall =20 min, while on foot (21.5 min), car (20 min) and other modes (20 min); median travel cost = 0 USD; median waiting time = 26.5 min, and consultation time = 10 min; median medical cost = 0 USD; travel time reflects a one-way journal

*** denotes significance at 1%, ** at 5%, and * at 10% level*** denotes significance at 1%, ** at 5%, and * at 10% level

## Abbreviations

ANC: Antenatal care
CI: Concentration index
DHS: Demographic health survey
ICHF: Improved Community Health Fund
LMICs: Low- and middle-income countries
MCH: Maternal and child health
MoHCDGEC: Ministry of Health, Community Development, Gender, Elderly and Children
NHIF: National Health Insurance Fund
P4P: Payment for performance
PHC: Primary health care
PHSDP: Primary Health Care Services Development Programme
PNC: Postnatal care
SHIB: Social Health Insurance Benefit
TZS: Tanzanian shilling
UHC: Universal health coverage
USD: United states dollar
